# COVID-19 infection characteristics, risk factors and its potential impacts on Takayasu arteritis: a web-based survey in a large cohort

**DOI:** 10.3389/fimmu.2023.1284168

**Published:** 2024-01-08

**Authors:** Xiufang Kong, Jinghua Wang, Guihua Fan, Huijing Huang, Ying Sun, Huiyong Chen, Lili Ma, Yanshan Li, Lindi Jiang

**Affiliations:** ^1^ Department of Rheumatology, Zhongshan Hospital, Fudan University, Shanghai, China; ^2^ Center of Clinical Epidemiology and Evidence-based Medicine, Fudan University, Shanghai, China; ^3^ Department of Rheumatology and Immunology, Linyi People’s Hospital, Linyi, Shandong, China

**Keywords:** Takayasu arteritis, COVID-19, characteristics, outcome, disease relapse

## Abstract

**Objectives:**

To investigate the characteristics of COVID-19 and its impact on patients with Takayasu’s arteritis (TAK).

**Methods:**

A web-based survey was administered to a TAK cohort and their co-residents in China during January 2023. Infection symptoms, post-acute sequelae of COVID-19 (PASC), potential impacts of COVID-19 on patients’ disease condition, treatment and immune-related parameters were analyzed. In addition, risk factors for COVID-19 and disease relapse after infection were explored.

**Results:**

The infection rate was significantly lower in patients with TAK than in co-residents (79.13% vs 90.67%, p=0.025). TAK patients were more prone to gastrointestinal symptoms (17.78% vs 5.88%, p=0.024), sleep problems (25.15% vs 10.29%, p=0.011), and symptoms involving more than 2 organs (58.90% vs 35.29%, p=0.001) after infection. Although only 2.45% of TAK patients were hospitalized and none progressed to life-threatening conditions, they were more likely to suffer from PASC (26.38% vs 13.24%, p=0.029), especially active patients. Active disease after the pandemic was significantly lower in infected patients than uninfected patients (21/163, 12.88% vs. 11/43, 25.58%, p=0.041). The presence of multiple system symptoms was a risk factor for active TAK after infection [OR: 3.62 (95% CI 1.06-12.31), p=0.040]. Moreover, csDMARDs treatment was a risk factor for COVID-19 infection [OR: 3.68 (95% CI 1.56-8.66), p=0.002].

**Conclusion:**

Although TAK patients with COVID-19 have more acute and post-acute symptoms, there is no adverse outcome and the risk of disease relapse does not increase. Patients treated with csDMARDs may be at higher risk of infection and deserve more clinical attention.

## Introduction

Takayasu’s arteritis (TAK) is a chronic inflammatory large-vessel vasculitis that primarily affects the aorta and its major branches (1). Although TAK mostly affects young or middle-aged women, it has been reported in people of virtually all ages and has a worldwide distribution (2).

As with other autoimmune rheumatic diseases (ARDs), the development of TAK is mediated by inflammation and autoimmunity, and it is commonly treated with glucocorticoids (GCs) and immunosuppressive medications (3, 4). However, these can increase the risk of microbial infection or exacerbate infection (5). The severe acute respiratory syndrome coronavirus 2 (SARS-CoV-2), responsible for COVID-19, has spread across the globe and infected over 760 million people worldwide (6). COVID-19 is now recognized as not only a lung condition but a multi-organ syndrome (7) which can also cause post-acute sequelae of COVID-19 (PASC) (8). It has been reported that patients with ARDs have higher rates of SARS-CoV-2 infection and an increased mortality rate (9). In addition, patients with ARDs often experience severe complications after infection due to immune imbalance, which can result in multiple organ damage (10). Moreover, COVID-19 might a risk factor for rheumatic disease relapse (11).

However, the prevalence and characteristics of COVID-19 in patients with TAK and its impact on TAK disease condition is still unclear. There are only two studies regarding COVID-19 in TAK patients. One study included 56 patients with TAK, but only 13 of them were infected with SARS-CoV-2. In addition, 95% of the infected patients had mild symptoms (12) and the impact of COVID-19 on patients’ relapse, immune parameters and treatment was not analyzed. Another study analyzed clinical features of COVID-19 in pediatric rheumatic disease, of which 5 patients had TAK (13). Unfortunately, their features and outcomes were not analyzed separately.

Therefore, this study aimed to explore potential impacts of COVID-19 on TAK based on a comprehensive investigation in a large cohort, including patients’ acute and post clinical symptoms, changes of disease conditions, immune functions, and treatment. Moreover, potential risk factors for patients’ COVID-19 infection and TAK relapse were explored.

## Methods

### Patients

This study was conducted with patients in the East China Takayasu Arteritis cohort, which was established by the Department of Rheumatology of Zhongshan Hospital in Shanghai, China. Patients in this cohort were mainly from Shanghai and the surrounding areas in the eastern region of China. All of them had been admitted to the hospital’s Department of Rheumatology and diagnosed with TAK in accordance with the criteria of the 1990 American College of Rheumatology (14). Patients’ clinical data were recorded in an electronic database. The study was approved by the Institutional Review Board of Zhongshan Hospital, Fudan University, China [approval number: B-2016-168(2)R] and it conforms to the provisions of the Declaration of Helsinki. Consent was obtained from all subjects before participating in this survey.

### Web-based survey of patients regarding COVID-19 infection

The study design is shown in **Supplementary Figure 1**. An electronic questionnaire was distributed to 465 patients through WeChat in January 2023. This survey was not anonymous and mainly investigated COVID-19 conditions from December 2022 to January 2023. The questionnaire surveyed four major areas: demographics, COVID-19 vaccination, COVID-19 infection, and TAK disease. The questions about COVID-19 infection included diagnosis method, symptoms (acute and chronic), duration, treatment, outpatient visit and hospitalization. Regarding TAK disease condition, patients’ symptoms and treatment before and after COVID-19 infection were surveyed. At the same time, TAK patients’ co-residents were asked to fill in another similar questionnaire without the TAK disease information.

### Clinical data collection and analysis

A total of 206 TAK patients and 75 co-residents responded to the questionnaire. Their clinical data including disease duration, disease activity, imaging types, organ involvement and treatment regimen before and after the pandemic were collected from our database. Active disease was defined as the National Institutes of Health score ≥ 2 points (15). Relapse was defined as recurrence of active disease (NIH score ≥ 2 points) after disease remission (4). Vascular imaging types were based on the 1996 Numano classification (16). Organ involvement was defined as organ dysfunction (e.g. heart/kidney failure, kidney atrophy) or ischemic event (e.g. brain/renal/lung infarction) due to TAK-related vascular stenosis or occlusion. In our study, PASC is defined as persistent COVID-19 symptoms, such as fatigue, dyspnea, memory impairment, insomnia, chest discomfort, arthralgia, anxiety or other injuries lasting over 30 days (17).

Laboratory results of TAK patients were collected within 3 months of both the last follow-up before COVID-19 infection and the first follow-up after infection. These parameters included erythrocyte sedimentation rate (ESR), C-reactive protein (CRP), immunoglobulin (Ig) G, IgM, IgA, interleukin-6, tumor necrosis factor α (TNF-α), complement 3 (C3), complement 4 (C4), CH50, and the percentages of different immune cells, such as the CD19+ B, CD3+ T, CD4+ T, CD8+ T and natural killer (CD56+) cells. These immune cells were detected by routine flow cytometry, including monocytes (CD14+), lymphocytes (CD3+), CD4+ T cells (CD3+CD4+), CD8+ T cells (CD3+CD8+), B cells (CD19+), and natural killer (NK) cells (CD56+). A panel of fluorochrome-labeled monoclonal antibodies including CD3 (FITC, BioLegend), CD4 (PE-Cy7, BioLegend), CD8 (PE-Texas Red, BioLegend), CD14 (BV510, BioLegend), CD19 (PerCP/Cyanine5.5, BioLegend), and CD56 (APC, BioLegend) were used in flow cytometry and detected by BDFACSAriaIII. All the tests were conducted at the central laboratory of our center. The ratio was calculated as the proportion of each subset relative to the total number of cells in the lymphocytes gate.

### Statistical analysis

Categorical data are expressed as numbers and percentages, continuous variables that are normally distributed are expressed as mean ± standard deviation (SD), while continuous variables with a skewed distribution are expressed as median and interquartile range. Parameters between the vaccinated and unvaccinated groups, infected and uninfected groups were compared. The χ^2^ test or Fisher’s exact test was used to analyze the categorical data. Student’s t-test or Wilcoxon-Mann-Whitney test was applied in continuous variables analysis, as appropriate. A binary regression analysis was used to identify risk factors of COVID-19 infection and TAK relapse after infection. SPSS 25.0 (IBM, Armonk, NY, USA) and Prism 9.1.0 (GraphPad, La Jolla, CA, USA) were used for the statistical analyses.

## Results

### Characteristics of COVID-19 infection in TAK patients

#### Patients with TAK have a lower infection rate but suffer more multi-systemic symptoms and PASC

The demographic and COVID-19-related characteristics of TAK patients (n=206) and co-residents (n=75) are shown in **Table 1**. Co-residents had a greater mean age and a higher BMI, and there was a lower proportion of non-smokers and females (all p<0.05, **Table 1**). Notably, significantly fewer TAK patients received at least 2 COVID-19 vaccinations than co-residents (42.23% vs. 76%, p<0.05, **Table 1**).

**Table 1:** Demographic and clinical characteristics of patients with TAK and their co-residents.

Characteristics	Patients with TAK (n=206)	Co-residents (n=75)	p
Age (mean ± SD, years)	35.83 ± 11.12	41.48 ± 12.98	**0.001**
Female, n (%)	179 (86.89)	36 (48)	**<0.001**
BMI (mean ± SD)	22.56 ± 3.83	23.74 ± 4.85	**0.048**
Smoker, n (%)	10 (4.85)	16 (21.33)	**<0.001**
Disease duration (median, IQR, months)	48 (24, 103)	/	/
COVID-19 vaccinated patients, n (%)	92 (44.66)	59 (78.67)	**<0.001**
≥2doses, n (%)	87 (42.23)	57 (76)	**<0.001**
**Imaging type, n (%)**		/	/
I	54 (26.21)		
IIA or IIB	33 (16.02)		
III	9 (4.37)		
IV	16 (7.77)		
V	92 (44.66)		
**TAK-associated organ involvement, n (%)**	86 (41.75)	/	/
Renal hypertension or renal atrophy	39 (18.93)		
Cerebral infarction	20 (9.71)		
Pulmonary hypertension	14 (6.80)		
Heart failure or cardiac infarction	22 (10.68)		
**TAK-associated treatment, n (%)**		/	/
GCs and biologic DMARDs	32 (15.53)		
GCs and JAK inhibitors	39 (18.93)		
GCs and conventional DMARDs	83 (40.29)		
Single GCs	22 (10.68)		
No treatment	30 (14.56)		

P, Comparison between the total TAK patients and their co-residents. Bold values indicate statistical significance between different groups.

Compared to the co-residents, patients with TAK had a lower rate of COVID-19 infection (79.13% vs 90.67%, p=0.025). Their infection characteristics are shown in **Table 2**. TAK patients were more prone to develop symptoms such as gastrointestinal symptoms (17.78% vs 5.88%, p=0.024), sleep problems (25.15% vs 10.29%, p=0.011), and had a higher chance of involvement of >2 organs (58.90% vs 35.29%, p=0.001). They also had higher frequencies of hospital visits (p=0.028), chest X-ray (p=0.012), and treatment with Chinese traditional medicine (p=0.029); but only 2.45% of patients with TAK were hospitalized and no patients progressed to life-threatening conditions.

**Table 2:** COVID-19 infection characteristics of patients with TAK and their co-residents.

Infection characteristics	Patients with COVID-19				Co-residents with COVID-19 (n=68)	p£
	Total (n=163)	Active disease& (n=21)	Inactive disease (n=142)	*p^§^*		
**Symptoms, n (%)**	**163 (79.13)**	**21 (65.63)**	**142 (81.61)**	**0.041**	**68 (90.67)**	**0.025**
Fever	129 (79.14)	14 (66.67)	115 (80.99)	0.152	55 (80.88)	0.764
Fatigue	109 (66.87)	17 (80.95)	92 (64.79)	0.142	43 (63.24)	0.595
Myalgia	92 (56.44)	17 (80.95)	75 (52.82)	**0.015**	36 (52.94)	0.626
Cough	128 (78.53)	17 (80.95)	111 (78.17)	1.00	46 (67.65)	0.080
Chest discomfort	29 (17.79)	9 (42.86)	20 (14.08)	**0.004**	9 (13.24)	0.395
Pharyngitis	51 (31.29)	8 (38.10)	43 (30.28)	0.471	24 (35.29)	0.553
Gastrointestinal symptoms	28 (17.78)	4 (19.05)	24 (16.90)	0.762	4 (5.88)	**0.024**
Dysosmia or anosmia	61 (37.42)	6 (28.57)	55 (38.73)	0.369	19 (27.94)	0.167
Joint pain	39 (23.93)	7 (33.33)	32 (22.54)	0.279	13 (19.12)	0.425
Headache	62 (38.04)	9 (42.86)	53 (37.32)	0.626	21 (30.88)	0.317
Sleep problems	41 (25.15)	6 (28.57)	35 (24.65)	0.699	7 (10.29)	**0.011**
More than 2 organs involved#	96 (58.90)	17 (80.95)	79 (55.63)	**0.028**	24 (35.29)	**0.001**
Treatment, n (%)						
Visit to hospital during infection	20 (12.27)	7 (33.33)	13 (9.15)	**0.006**	2 (2.94)	**0.028**
Hospitalization	4 (2.45)	2 (9.52)	2 (1.41)	0.081	0 (0)	0.323
Chest X-ray	13 (7.98)	4 (19.05)	9 (6.34)	0.067	0 (0)	**0.012**
X-ray confirmed pneumonia	9 (69.23)	4 (19.05)	5 (55.56)	0.228	/	
Antipyretic drug treatment	85 (52.15)	12 (57.14)	73 (51.41)	0.623	38 (69.09)	0.604
Chinese traditional medicine	37 (22.70)	5 (23.81)	32 (22.54)	1.00	7 (10.29)	**0.029**
Duration time (median, IQR, days)	7 (6, 10)	8 (5, 9.75)	7 (6, 10)	0.746	7 (7, 9)	0.410
**PASC, n (%)**	43 (26.38)	12 (57.14)	31 (21.83)	**0.001**	9 (13.24)	**0.029**
Fatigue	22 (13.50)	11 (52.38)	11 (7.75)	**<0.001**	7 (10.29)	0.66
Cough	28 (17.18)	8 (38.09)	20 (14.08)	**0.01**	3 (4.41)	**0.01**
Anxiety	8 (4.91)	2 (9.52)	6 (4.23)	0.27	0 (0)	0.11
Numbness	3 (1.84)	3 (14.28)	0 (0)	**0.002**	0 (0)	0.56
Sleep problems	14 (8.59)	3 (14.29)	11 (7.75)	0.39	2 (2.94)	0.16
Palpitation	14 (8.59)	6 (28.57)	8 (5.63)	**0.003**	1 (1.47)	0.07
Dysosmia or anosmia	9 (5.52)	1 (4.76)	8 (5.63)	1.00	1 (1.47)	0.29
More than 2 organs involvement	21 (12.88)	4 (19.05)	17 (11.97)	0.48	2 (2.94)	**0.03**

&Disease activity was evaluated according to Kerr criteria within three months after COVID-19 infection. #The patients also suffered from systemic symptoms, such as fever, fatigue, and/or myalgia; £Comparison between the total TAK patients and their co-residents; §Comparison between TAK patients with and without active disease. PASC is defined as persistent COVID-19 symptoms, such as fatigue, dyspnea, memory impairment, insomnia, chest discomfort, arthralgia, anxiety or other injuries lasting over 30 days. Bold values indicate statistical significance between different groups.

**Figure 1:**
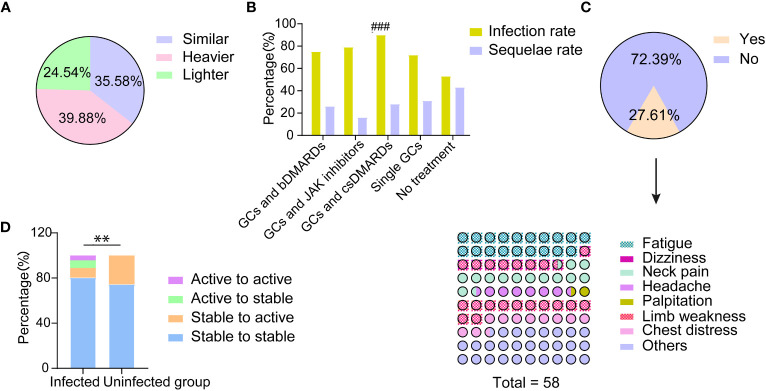
The impact of COVID-19 on the disease condition of patients with TAK. **(A)** Patients’ self-reported assessment regarding COVID-19 symptoms compared with the general population; **(B)** The incidence of COVID-19 infection and the rate of post COVID-19 syndrome in patients with different treatments after infection; ###significant difference in infection rate (p<0.001); **(C)** Patients’ self-reported major aggravating TAK-associated symptoms and their exacerbation after COVID-19 infection; **(D)** The disease condition changes of patients with and without COVID-19 infection before and after the pandemic; **significant difference in the proportion of patients changing from stable to active between those with and without infection (p<0.01).

About 40% of TAK patients reported that their COVID-19 symptoms were more severe than the general population, 24% were milder and 36% were similar (**Figure 1A**). TAK patients were more likely to have PASC (26.38% vs 13.24%, p=0.029), although there was no significant difference in the incidence of specific sequelae (**Table 2**).

We found that patients with active disease within three months before infection had a higher hospital visit rate (p=0.006), and suffered more multi-systemic damage (p=0.028), myalgia (0.015), and chest discomfort (p=0.004). The incidence of PASC was also higher in these patients than in patients without active disease (p=0.001, **Table 2**).

#### Vaccinated patients have less frequency of fever than unvaccinated patients

Of the 206 patients, 92 (44.66%) received at least one COVID-19 vaccination, which was lower than that in co-residents (p<0.001, **Table 1**). However, the infection rate was significantly higher in vaccinated patients than unvaccinated patients (79/92, 85.86% vs. 84/114, 73.68%, p=0.03). There were also some differences in the infection symptoms between the vaccinated and unvaccinated patients (**Supplementary Table 1**). Specifically, the vaccinated patients had less frequency of fever, but more anxiety than the unvaccinated patients after COVID-19 infection (69.62% vs. 88.10%, p=0.004; 33.33% vs. 4.35%, p=0.019, respectively).

### Patients treated with GCs and csDMARDs may have higher risks of infection

The clinical features of TAK patients with and without infection are reported in **Table 3**. No differences were observed in their demographic and clinical features except for smoking, which was significantly higher in the uninfected group (p=0.035, **Table 3**).

**Table 3:** Comparison of clinical characteristics between TAK patients with and without COVID-19 infection.

Characteristics	Infected group (n=163)	Uninfected group (n=43)	*p*
Age (mean ± SD, years)	36.48 ± 10.83	33.37 ± 11.97	0.080
Female, n (%)	145 (88.96)	34 (79.07)	0.087
BMI (mean ± SD)	22.81 ± 4.04	21.60 ± 2.73	0.176
Smoker, n (%)	5 (3.07)	5 (11.63)	**0.035**
Disease duration (median, IQR, months)	52.5 (24, 108)	45 (24, 84)	0.438
Active disease&, n (%)	18 (11.04)	0 (0)	**0.016**
Imaging type, n (%)			
I	43 (26.38)	11 (25.58)	1
IIA or IIB	26 (15.95)	7 (16.28)	1
III	6 (3.68)	3 (6.98)	0.399
IV	12 (7.36)	4 (9.30)	0.749
V	74 (45.40)	18 (41.86)	0.678
TAK-associated ischemic event, n (%)	65 (39.88)	21 (48.84)	0.289
Renal hypertension or renal atrophy	29 (17.79)	10 (23.26)	0.416
Cerebral infarction	15 (9.20)	5 (11.63)	0.575
Pulmonary hypertension	11 (6.75)	3 (6.98)	1
Heart failure or cardiac infarction	18 (11.04)	4 (9.30)	1
**Major treatment**			**0.001**
GCs and biologic DMARDs, n (%)	24 (14.72)	8 (18.60)	0.532
TCZ	3 (1.84)	0 (0)	1
ADA	9 (5.52)	5 (11.63)	0.175
IL-17Ab	10 (6.14)	5 (11.63)	0.318
GCs and JAK inhibitors, n (%)			
TOF	31 (19.02)	8 (18.60)	1
GCs and conventional DMARDs, n (%)	75 (46.01)	8 (18.60)	**0.001**
LEF	38 (23.31)	0 (0)	**<0.001**
MTX	14 (8.59)	6 (13.95)	0.383
MMF	6 (3.68)	1 (2.33)	1
AZA	4 (2.45)	0 (0)	0.582
CTX	3 (1.84)	0 (0)	1
HCQ	13 (7.98)	2 (4.65)	0.742
Others^¥^	6 (3.68)	0 (0)	0.348
Single GCs	17 (10.43)	5 (11.63)	0.785
No treatment	16 (9.82)	14 (32.56)	**<0.001**

&Disease activity was evaluated according to Kerr criteria within three months before COVID-19 infection. ¥ including azathioprine, cyclosporin, tacrolimus, thalidomide, and iguratimod. Bold values indicate statistical significance between different groups.

However, there was a difference in TAK treatments between the infected and uninfected groups (**Table 3**, **Figure 1B**). The proportion of patients treated with GCs and csDMARDs in the infected group (46.01%) was significantly higher than in the uninfected group (18.60%, p=0.001, **Table 3**). Leflunomide was most commonly used in these patients (23.31%), which was not found in patients without COVID-19. Conversely, the proportion of patients without any treatment was lower in the infected group (9.82% vs 32.56%, p<0.001, **Table 3**).

The infection rate in patients with different treatments was successively analyzed (**Figure 1B**). Patients treated with GCs and csDMARDs had the highest rate of COVID-19 infection (90.36%), which was significantly higher than in patients without any treatment (p<0.001, **Figure 1B**). No significant differences were found in demographic or other clinical features among patients with different treatments (**Supplementary Table 2**). The logistic regression analysis indicated that patients treated with GCs and csDMARDs was more likely to be infected with COVID-19 (OR=3.68 [95% CI 1.56-8.66], p=0.003, **Table 4**).

**Table 4:** The binary regression analysis for the risk of COVID-19 infection among patients with TAK or risk of active disease after COVID-19 infection among infected patients.

Risk factors	Crude model			Adjusted model		
	OR	95% CI	p	OR	95% CI	p
For COVID-19 infection#						
Smoker	0.30	0.07-1.17	0.08	0.38	0.09-1.59	0.19
BMI	1.12	1.002-1.26	**0.046**	1.11	0.99-1.25	0.07
GCs and csDMARDs treatment	3.85	1.64-9.02	**0.002**	3.68	1.56-8.66	**0.003**
For active disease after COVID-19 infection*						
Multiple system involvement	4.13	1.22-13.94	**0.022**	3.62	1.06-12.31	**0.040**
Post COVID-19 syndrome	0.32	0.08-1.21	**0.091**	0.47	0.14-1.54	0.211
Visit to hospital	0.14	0.04-0.48	**0.002**	0.14	0.04-0.47	**0.002**

#The different factors found between the infected and uninfected group in **Table 3** were included here (the factor active disease was not included because there was no patient with active disease in the uninfected group).

*The different factors found between the active and inactive groups in TAK patients with COVID-19 infection (**Table 1**) were included here.

Data are presented as ORs with 95% CIs, and p values. All adjusted ORs have been adjusted for age and sex. Bold values indicate statistical significance between different groups.

### Potential impacts of COVID-19 infection on patients’ disease condition

According to the survey, 27.60% (45/163) of TAK patients with COVID-19 reported that their TAK-associated symptoms worsened after infection (**Figure 1C**). The major aggravating symptoms were weakness and neck pain (**Figure 1C**).

#### Changes of disease condition in pre-active TAK patients after COVID-19 infection

Eighteen out of 206 (8.74%) TAK patients relapsed before the COVID-19 pandemic. Of these, 7 (38.89%) of them were still flaring after COVID-19 infection, but there was no difference in COVID-19 clinical features between patients who continuously relapsed and those who achieved remission (**Supplementary Table 3**).

#### COVID-19 infection does not increase risk of disease relapse in patients with TAK

Fourteen infected and 11 uninfected patients developed new disease relapse, respectively, after the pandemic. There was no difference in demographic, clinical characteristics and lab tests (**Supplementary Table 4**). However, the rate of GCs and csDMARD treatment was significantly higher in infected patients (p=0.028, **Supplementary Table 4**).

As a whole, the rate of active disease in patients with COVID-19 was significantly lower than in uninfected patients (21/163, 12.88% vs. 11/43, 25.58%, p=0.041). The proportion of patients with disease relapse (stability to activity) was also lower in patients with COVID-19 compared to patients without (14/163, 8.59% vs. 11/43, 25.58%, p=0.002, **Figure 1D**).

According to the differential factors in **Table 2**, multiple system involvement, hospital visit and PASC were included in the regression model. The results showed that TAK patients with multiple system involvement after infection was a risk factor for disease relapse or un-remission after adjusting for age and gender [OR: 3.62 (95% CI 1.06-12.31), p=0.040, **Table 4**]. Notably, a hospital visit seemed to help avoid a TAK relapse after infection [OR: 0.14 (95% CI 0.04-0.47), p=0.002, **Table 4**].

#### Almost half of patients stop bDMARDs or JAK inhibitors temporarily during infection

**Figure 2:**
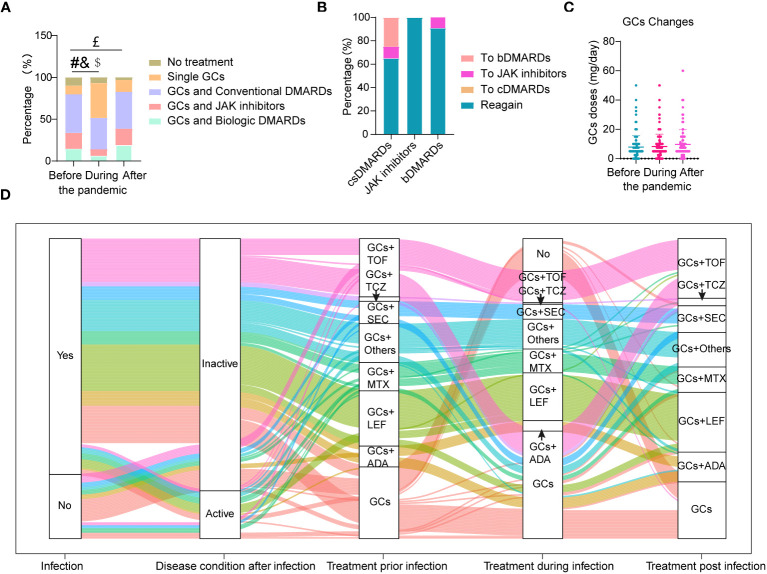
Changes in drugs used to treat TAK during COVID-19 infection. **(A)** Proportion of different drugs used before, during and after COVID-19 infection; **(B)** Conversion rate between different drugs after COVID-19 infection; **(C)** The highest doses of glucocorticoids (GCs) per day used before, during and after COVID-19 infection; **(D)** The whole treatment change pattern of TAK patients either with or without COVID-19 infection (n=190); patients without treatment were not listed here (n=16). #Significant difference in the proportion of patients on GCs and biologic DMARDs before and during infection (p<0.05); &Significant difference in the proportion of patients on GCs and JAK inhibitors before and during infection (p<0.05); $Significant difference in the proportion of patients on single GCs before and during infection (p<0.05); £Significant difference in the proportion of patients without treatment before and after infection (p<0.05).

The changes of drugs used to treat TAK during COVID-19 infection can be seen in **Figure 2**. During COVID-19 infection, the proportion of patients treated with bDMARDs and JAK inhibitors were significantly decreased, accompanied with the increase of patients with GCs treatment alone (all p<0.05, **Figure 2A**). The use of bDMARDs and JAK inhibitors decreased by 45.83% (10/24) and 48.39% (15/31), respectively (**Figure 2A**). When they recovered, more than 90% of them had returned to the original medications or doses (**Figure 2B**). In contrast, part of the patients who stopped csDMARDs had switched to JAK inhibitors (10%, 2/14) or bDMARDs (21.43%, 3/14) (**Figure 2B**). Among these patients, 2 of them changed their treatment regimen due to disease relapse. As a whole, the ratio of patients without treatment was decreased after treatment (p<0.05, **Figure 2A**).

The percentages of patients who stopped, increased, decreased or did not change use of GCs during infection were 3.23%, 7.10%, 1.94%, and 87.74%, respectively. There was no change in GC doses before, during and after COVID-19 infection (**Figure 2C**). Treatment between patients with and without disease activity after COVID-19 infection (**Supplementary Table 5**) did not change except for an increase of GCs in patients with disease activity. The whole treatment changes for TAK patients either with or without COVID-19 infection are shown in **Figure 2D**.

#### Changes of immune-related parameters in TAK patients after COVID-19 infection

**Figure 3:**
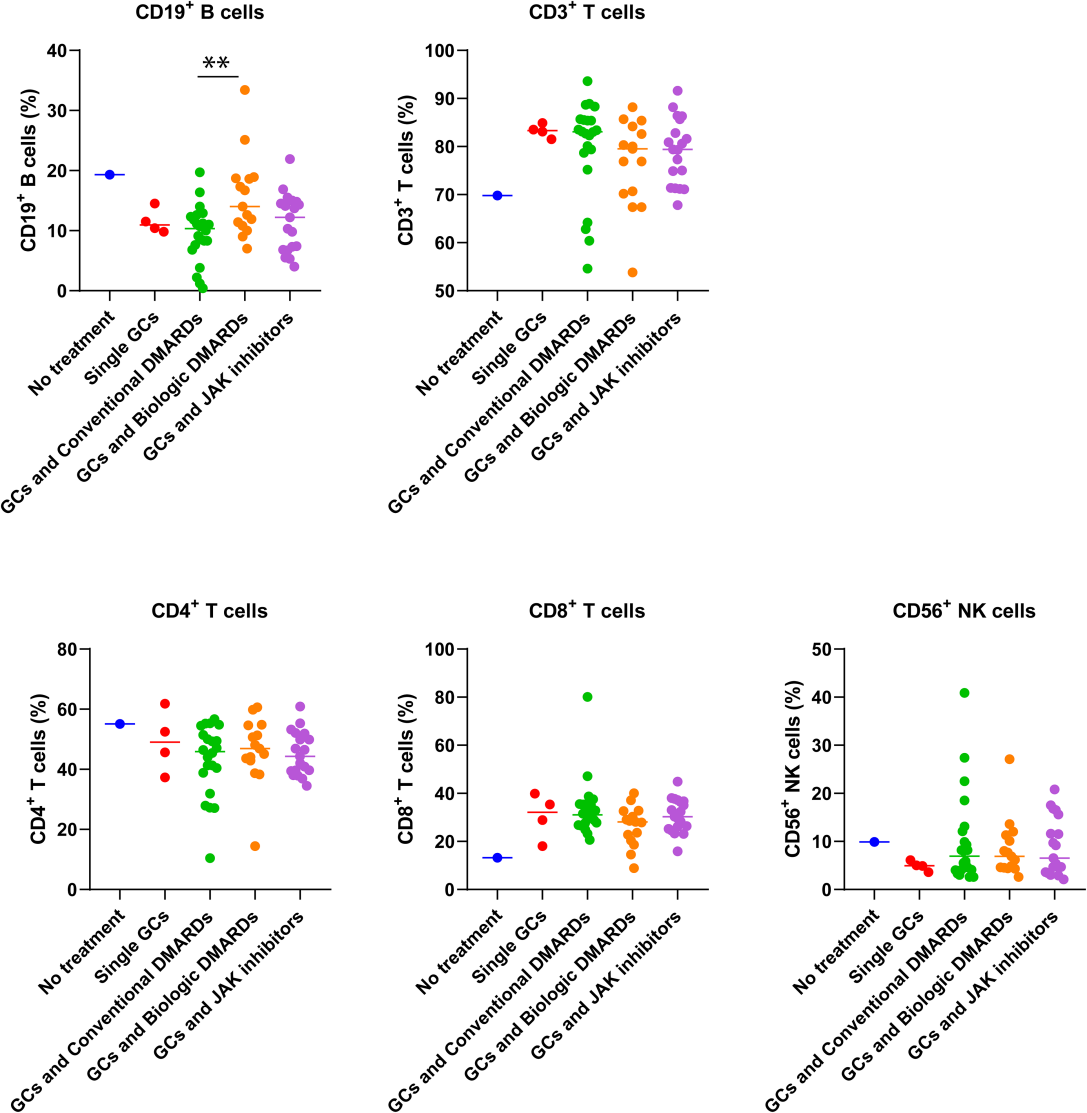
Differences in immune cell percentages of TAK patients with different treatments before the COVID-19 pandemic. ** p<0.01, comparison of percentage of CD19+B cells between patients with conventional DMARDs and biologic DMARDs. The number of patients without treatment, with single GCs, conventional DMARDs, biologic DMARDs, JAK inhibitors were 1, 4, 22, 15, and 19 respectively.

The imbalance of immune functions might lead patients in risk of COVID-19 (18). TAK patients on GCs and csDMARDs had the highest rate of COVID-19 infection (**Figure 1B**). As expected, they had lowest CD19^+^ B cell ratio among different treatment groups, which was significantly lower than that in patients on GCs and bDMARDs (p=0.006, **Figure 3**). No differences in B cell ratios between patients on GCs and csDMARDs and patients on GCs and tsDMARDs (p = 0.23, **Figure 3**) or patients treated with GCs alone (p = 0.42, **Figure 3**). However, compared to the other groups as a whole, the GC and csDMARDs group still had a significantly lower proportion of B cells (9.54 ± 4.72 vs. 13.25 ± 5.83, p = 0.01). No differences in other immune parameters were found between different treatment groups.

Changes of these parameters were analyzed before and after COVID-19 infection (**Supplementary Figure 2**). The results indicated significant decreases in IgM (p=0.01), CH50 (p=0.04) and CD19^+^ B cells (p=0.03), but no changes were found in IgA, IgG, C3, C4, CD3^+^ T cells, CD4^+^ T cells, CD8^+^ T cells, or CD56^+^ NK cells.

## Discussion

This study analyzed the characteristics of COVID-19 infection in patients with TAK as well as potential impacts on patients’ disease activity, treatment and immune-related parameters. Although patients had a lower infection rate compared with their co-residents, they were more likely to develop multi-systemic symptoms and PASC. However, we didn’t find serious adverse outcomes and increased risk of disease relapse. However, we found that patients treated with GCs and csDMARDs may be susceptible to infection due to a relatively lower percentage of CD19+ B cells.

In this study, patients’ co-residents were surveyed as the control population. In this way, some confounding factors related with infection such as exposure environment or variant of SARS-CoV-2 might be controlled to some extent. The lower infection rate in patients with TAK was possibly due to their stricter self-protection measures. Consistent with this phenomenon, a relatively low incidence of COVID-19 infection has also been reported in other rheumatic diseases (19, 20).

Regarding the infection symptoms, TAK patients had a higher likelihood of having multi-system symptoms, which was also similar to other studies (21, 22). However, the risk of developing adverse outcomes was low. Different from this, an increase of ischemic events in giant cell arteritis (GCA) has been reported during pandemic in Italy, which was largely due to failure to receive timely medical care (23). Some other studies have also reported risks of developing serious outcomes in people with rheumatic diseases after COVID-19 infection (24–26). Moreover, the COVID-19 Global Rheumatology Alliance Vaccine Survey found that about 25% of patients with ARDs had PASC (27), which was similar to our study (26.38%). Therefore, rheumatologists and patients should be more cautious after infection.

Currently, the evidence regarding TAK relapse rate after COVID-19 infection is limited. In our study, less than 10% patients with TAK developed new-onset relapses, which was even lower than that in patients without infection. Regarding other rheumatic disease, the results from different studies were not conclusive. One study observed that disease activity of rheumatoid arthritis and spondyloarthritis was unchanged after COVID-19 infection (28). Another prospective study reported a 41% flare rate in patients with ARDs after COVID-19 (29). Overall, COVID-19 may have different effects on different rheumatic diseases. It is suggested to increase the follow-up frequency of TAK patients to avoid potential side effects during and post the epidemic.

GCs and immunosuppressants are routinely used to control TAK (4), resulting in a suppressed immune state. As expected, in our study, the infection rate was higher in patients with immunosuppressants than that in patients without treatment. Consistent with this result, Shen et al. reported that individuals treated with immunosuppressants had a higher risk of infection (30, 31). However, we found that patients treated with csDMARDs rather than b/tsDMARDs might be at higher risk for COVID-19 in TAK patients. Consistently, some studies reported that COVID-19 infection rate was low in ARDs patients on bDMARDs or tsDMARDs (32, 33). This might relate with their different pharmacological effects on coronavirus infections (34). In line with this, we found that the percentage of CD19^+^ B cells was lowest in patients on GCs and csDMARDs. As B cells play a fundamental role in the prevention of COVID-19 (35), whether the low proportion of B cells is associated with a high rate of infection in these patients requires further validation.

The limitations of this study included several aspects. First, this survey was retrospectively conducted, there might be response and recall bias among different populations. Secondly, there were several differences between the diseased group and control group such as gender, age, etc. In addition, the sample size in some subgroup was small, such as patient without treatment in immune cell analysis. At last, whether csDMARDs increases COVID-19 infection risk and whether this is related with B cells reduction requires additional exploration.

In conclusion, this is the first study to comprehensively analyze COVID-19 infection characteristics in TAK. Although patients with TAK have more symptoms and PASC after infection, there is no adverse outcome and the risk of disease relapse does not increase. Patients treated with csDMARDs may be at higher risk of infection and deserve more clinical attention.

## Data availability statement

The original contributions presented in the study are included in the article/**Supplementary Material**. Further inquiries can be directed to the corresponding authors.

## Ethics statement

The studies involving humans were approved by Institutional Review Board of Zhongshan Hospital, Fudan University. The studies were conducted in accordance with the local legislation and institutional requirements. The participants provided their written informed consent to participate in this study.

## Author contributions

XK: Formal Analysis, Writing – original draft, Funding acquisition. JW: Data curation, Validation, Project administration, Investigation, Writing – review & editing. GF: Data curation, Investigation, Methodology, Project administration, Formal Analysis, Writing – review & editing. HH: Data curation, Investigation, Methodology, Project administration, Formal Analysis, Writing – review & editing. YS: Investigation, Resources, Methodology, Project administration, Formal Analysis, Writing – review & editing. HC: Data curation, Validation, Investigation, Project administration, Formal Analysis, Writing – review & editing. LM: Data curation, Validation, Investigation, Methodology, Project administration, Formal Analysis, Writing – review & editing. YL: Formal Analysis, Writing – review & editing, Investigation. LJ: Conceptualization, Project administration, Supervision, Funding acquisition, Investigation, Writing – review & editing.
