# Supplementary material for: COVID-19 infection characteristics, risk factors and its potential impacts on Takayasu arteritis: a web-based survey in a large cohort
Source: Front Immunol. 2024 Jan 8;14:1284168. doi: 10.3389/fimmu.2023.1284168 (PMC10800358; doi:10.3389/fimmu.2023.1284168)
Supplement: Supplementary file 1 [file DataSheet_1.docx]

**Supplementary Figure S1. Study design**


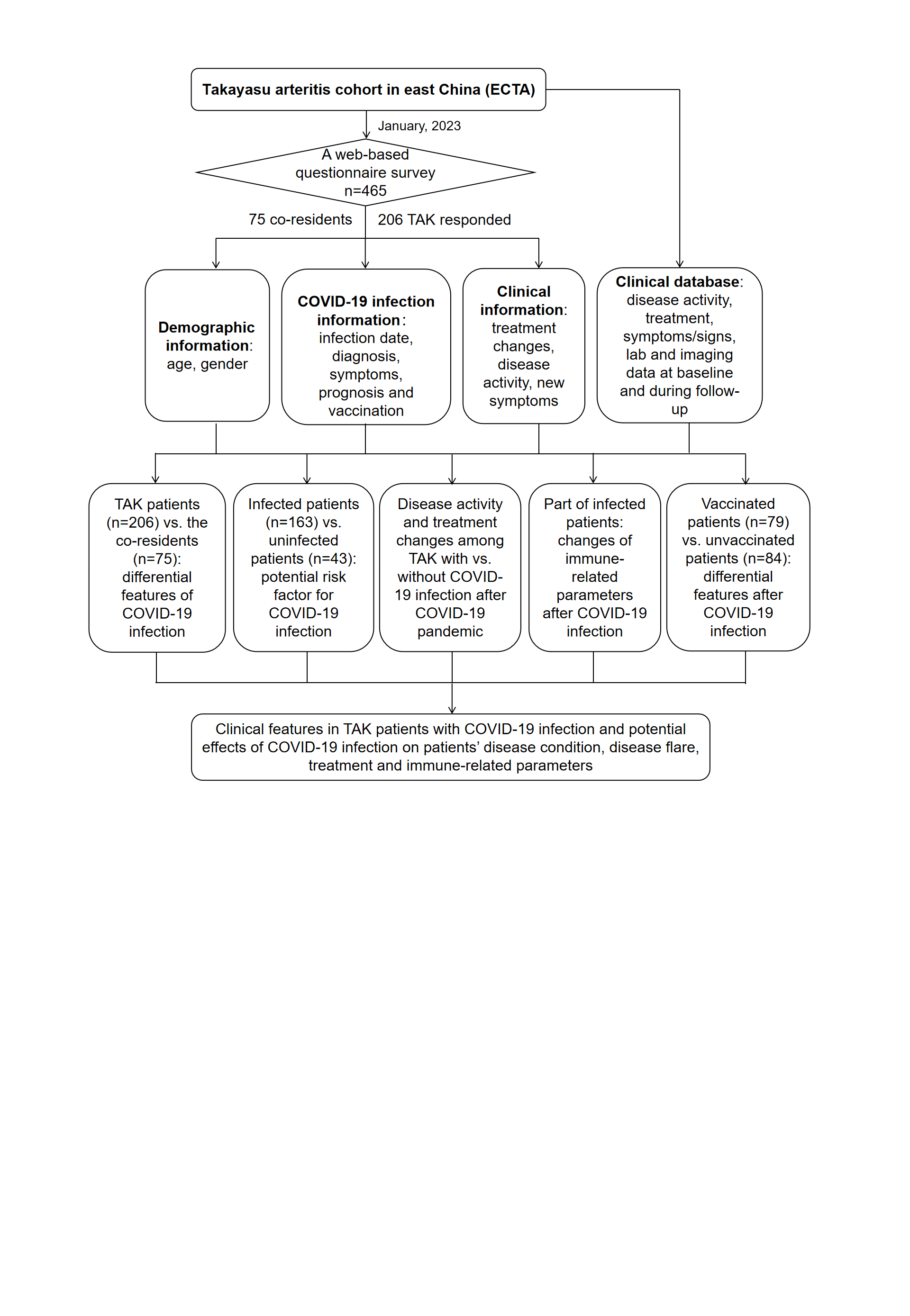


**Supplemental Table S1**. The clinical features of Takayasu’s arteritis patients with and without COVID-19 vaccination after infection

| **Characteristics** | **Vaccinated group**  **(n=79)** | **Unvaccinated group**  **(n=84)** | ***p*** |
| --- | --- | --- | --- |
| Age (mean±SD, years) | 37.13±10.90 | 35.87±10.80 | 0.460 |
| Female, n (%) | 68 (86.08) | 77 (91.67) | 0.255 |
| BMI (mean±SD) | 22.72±4.31 | 22.90±3.80 | 0.414 |
| Disease duration (median, IQR, months) | 43 (11.75, 96) | 60 (36.25, 108) | **0.009** |
| Disease activity&, n (%) | 12 (15.19) | 6 (7.14) | 0.101 |
| Imaging type, n (%) |  |  |  |
| I | 21 (26.58) | 22 (26.19) | 1 |
| IIA or IIB | 14 (17.72) | 12 (14.29) | 0.549 |
| III | 1 (1.27) | 5 (5.95) | 0.211 |
| IV | 9 (11.39) | 3 (3.57) | 0.023 |
| V | 34 (43.04) | 40 (47.62) | 0.557 |
| TAK-associated organ involvement, n (%) | 38 (48.10) | 35 (41.67) | 0.409 |
| Renal hypertension or renal atrophy | 18 (22.78) | 11 (13.10) | 0.106 |
| Cerebral infarction | 8 (10.13) | 7 (8.33) | 0.692 |
| Pulmonary hypertension | 5 (6.33) | 6 (7.14) | 1 |
| Heart failure or cardiac infarction | 7 (8.86) | 11 (13.10) | 0.389 |
| Major treatment, n (%)* |  |  |  |
| GCs and bDMARDs | 11 (13.92) | 13 (15.48) | 0.99 |
| GCs and JAK inhibitors | 15 (18.99) | 16 (19.05) |  |
| GCs and csDMARDs | 36 (45.57) | 39 (46.43) |  |
| Single GCs | 9 (11.39) | 8 (9.52) |  |
| No treatment | 8 (10.13) | 8 (9.52) |  |
| Medication changes during COVID-19 infection, n (%)* |  |  |  |
| GCs | 11 (15.49) | 8 (10.53) | 0.37 |
| bDMARDs | 4 (36.36) | 7 (53.85) | 0.392 |
| JAK inhibitors | 4 (26.67) | 11 (68.75) | **0.019** |
| csDMARDs | 7 (19.44) | 13 (33.33) | 0.174 |
| COVID-19 symptom, n (%) |  |  |  |
| Fever | 55 (69.62) | 74 (88.10) | **0.004** |
| Highest body temperature (mean ± SD, ℃） | 38.89±0.79 | 38.88±0.64 | 0.817 |
| Fatigue | 52 (65.82) | 57 (67.86) | 0.783 |
| Myalgia | 41 (51.90) | 51 (60.71) | 0.257 |
| Cough | 65 (82.28) | 63 (75) | 0.258 |
| Chest discomfort | 12 (15.19) | 17 (20.24) | 0.4 |
| Pharyngitis | 26 (32.91) | 25 (29.76) | 0.665 |
| Gastrointestinal symptoms | 10 (12.66) | 18 (21.43) | 0.138 |
| Dysosmia or anosmia | 32 (40.51) | 30 (35.71) | 0.529 |
| Joint pain | 21 (26.58) | 18 (21.43) | 0.441 |
| Headache | 30 (37.97) | 32 (38.10) | 1 |
| Sleep problems | 17 (21.52) | 24 (28.57) | 0.3 |
| More than 2 organs involved# | 47 (59.49) | 49 (58.33) | 1 |
| Prognosis of COVID-19 infection, n (%) |  |  |  |
| Visit to hospital during infection | 10 (12.66) | 10 (11.90) | 1 |
| Hospitalization | 2 (2.53) | 2 (2.38) | 1 |
| Chest X-ray | 6 (7.59) | 7 (8.33) | 1 |
| X-ray confirmed pneumonia | 3 (50) | 6 (85.71) | 0.266 |
| Antipyretic drugs treatment | 37 (46.84) | 48（57.14） | 0.188 |
| Chinese traditional medicine | 16 (20.25) | 21 (25) | 0.47 |
| Duration of COVID-19 infection (median, IQR, days) | 7 (5, 10) | 7 (7, 10) | 0.080 |
| Remaining symptoms after 30 days of COVID-19 infection, n (%) | 21 (26.58) | 23 (27.38) | 1 |
| Fatigue | 10 (47.62) | 12 (52.17) | 0.763 |
| Cough | 16 (76.19) | 12 (52.17) | 0.098 |
| Anxiety | 7 (33.33) | 1 (4.35) | **0.019** |
| Numbness | 2 (9.52) | 1 (4.35) | 0.599 |
| Sleep problems | 7 (33.33) | 7 (30.43) | 1 |
| Palpitation | 6 (28.57) | 8 (34.78) | 0.659 |
| Dysosmia or anosmia | 3 (14.29) | 6 (26.09) | 0.462 |
| More than 2 organs involved | 9 (42.86) | 12 (52.17) | 0.537 |

^&^Disease activity was evaluated according to Kerr criteria three months before COVID-19 infection. #The patients also suffered systemic symptoms, such as fever, fatigue, and/or myalgia. *Major treatment or medical changes. GCs, glucocorticoids; bDMARDs, biologic disease-modifying anti-rheumatic drugs; csDMARDs, conventional synthetic disease-modifying anti-rheumatic drugs.

**Supplemental Table S2.** Clinical characteristics of Takayasu’s arteritis patients with COVID-19 infection on different treatments.

| **Characteristics** | **No treatment** | **Single GCs** | **GCs and csDMARDs** | **GCs and JAK inhibitors** | **GCs and bDMARDs** | **p** |
| --- | --- | --- | --- | --- | --- | --- |
| N | 16 | 17 | 75 | 31 | 24 |  |
| Age (mean±SD, years) | 37.06±14.01 | 39.35±12.11 | 37.57±11.80 | 32.87±7.45 | 35.29±6.85 | 0.232 |
| Female, n (%) | 13 (81.25) | 13 (76.47) | 70 (93.33) | 27 (87.10) | 22 (91.67) | 0.189 |
| BMI (mean±SD) | 22.82±3.46 | 22.98±3.48 | 22.69±4.41 | 23.50±4.57 | 22.19±2.83 | 0.796 |
| Disease duration (median, IQR, months) | 36 (10.50, 59.50) | 72 (21, 174) | 72 (39.25, 120) | 41 (24, 60) | 34 (11.75, 69) | 0.283 |
| Disease activity&, n (%) | 3 (18.75) | 1 (5.88) | 5 (6.67) | 4 (12.90) | 5 (20.83) | 0.209 |
| Smoker, n (%) | 1 (6.25) | 1 (5.88) | 0 (0) | 2 (6.45) | 1 (4.17) | 0.071 |
| Imaging type, n (%) |  |  |  |  |  |  |
| I | 3 (18.75) | 1 (5.88) | 24 (32) | 6 (19.35) | 9 (37.50) | 0.127 |
| IIA or IIB | 4 (25) | 3 (17.65) | 13 (17.33) | 3 (9.68) | 3 (12.50) | 0.609 |
| III | 1 (6.25) | 2 (11.76) | 2 (2.67) | 1 (3.23) | 0 (0) | 0.233 |
| IV | 0 (0) | 1 (5.88) | 6 (8) | 5 (16.13) | 0 (0) | 0.193 |
| V | 7 (43.75) | 9 (52.94) | 30 (40) | 16 (51.61) | 12 (50) | 0.678 |
| TAK-associated organ involvement, n (%) | 6 (37.50) | 6 (35.29) | 29 (38.67) | 13 (41.94) | 11 (20.37) | 0.958 |
| Renal hypertension or renal atrophy | 3 (18.75) | 2 (11.76) | 12 (16) | 9 (29.03) | 3 (12.50) |  |
| Cerebral infarction | 1 (6.25) | 1 (5.88) | 6 (8) | 1 (3.23) | 6 (25) |  |
| Pulmonary hypertension | 2 (12.5) | 1 (5.88) | 7 (9.33) | 1 (3.23) | 0 (0) |  |
| Heart failure or cardiac infarction | 2 (12.5) | 0 (0) | 12 (16) | 1 (3.23) | 3 (12.50) |  |
| COVID-19 associated symptoms |  |  |  |  |  |  |
| Fever, n (%) | 14 (87.50) | 15 (88.24) | 58 (77.33) | 26 (83.87) | 16 (66.67) | 0.421 |
| Highest body temperature (mean±SD, ℃） | 38.72±0.64 | 38.69±0.57 | 38.92±0.78 | 38.96±0.70 | 38.87±0.61 | 0.073 |
| Fatigue, n (%) | 8 (50) | 9 (52.94) | 57 (76) | 19 (61.29) | 16 (66.67) | 0.147 |
| Myalgia, n (%) | 8 (50) | 8 (47.06) | 43 (57.33) | 19 (61.29) | 14 (58.33) | 0.872 |
| Cough, n (%) | 9 (56.25) | 12 (70.59) | 60 (80) | 28 (90.32) | 19 (79.17) | 0.094 |
| Chest discomfort, n (%) | 2 (12.50) | 2 (11.76) | 12 (16) | 7 (22.58) | 6 (25) | 0.729 |
| Pharyngitis, n (%) | 7 (43.75) | 3 (17.65) | 21 (28) | 15 (48.39) | 5 (20.83) | 0.077 |
| Gastrointestinal symptoms, n (%) | 2 (12.50) | 1 (5.88) | 12 (16) | 7 (22.58) | 6 (25) | 0.496 |
| Dysosmia or anosmia, n (%) | 7 (43.75) | 6 (35.29) | 29 (38.67) | 12 (38.71) | 7 (29.17) | 0.898 |
| Joint pain, n (%) | 3 (18.75) | 3 (17.65) | 19 (25.33) | 10 (32.26) | 4 (16.67) | 0.691 |
| Headache, n (%) | 4 (25) | 4 (23.53) | 29 (38.67) | 14 (45.16) | 11 (45.83) | 0.410 |
| Sleep problems, n (%) | 3 (18.75) | 5 (29.41) | 19 (25.33) | 7 (22.58) | 6 (25) | 0.979 |
| More than 2 organs involved#, n (%) | 6 (37.50) | 9 (52.94) | 48 (64) | 20 (64.52) | 13 (54.17) | 0.319 |
| Prognosis of COVID-19 infection, n (%) |  |  |  |  |  |  |
| Visit to hospital during infection | 3 (18.75) | 1 (5.88) | 13 (17.33) | 1 (3.23) | 2 (8.33) | 0.225 |
| Chest X-ray | 2 (12.50) | 1 (5.88) | 8 (10.67) | 1 (3.23) | 1 (4.17) | 0.941 |
| Antipyretic drugs treatment | 9 (56.25) | 8 (47.06) | 37 (49.33) | 19 (61.29) | 11 (45.83) | 0.954 |
| Chinese traditional medicine | 1 (6.25) | 9 (52.94) | 15 (20) | 5 (16.13) | 7 (29.17) | 0.168 |
| Duration of COVID-19 positive (median, IQR, months) | 7 (5, 8) | 6.50 (3.25, 11) | 7 (6, 10) | 8 (7, 10) | 8 (7, 11.5) | 0.289 |
| Remaining symptoms after 30 days of COVID-19 infection, n (%) | 6 (37.50) | 4 (23.53) | 21 (28) | 5 (16.13) | 7 (29.17) | 0.551 |
| Fatigue | 4 (66.67) | 1 (25) | 13 (61.90) | 2 (40) | 2 (28.57) | 0.792 |
| Cough | 2 (33.33) | 3 (75) | 15 (71.43) | 3 (60) | 5 (71.43) | 0.600 |
| Anxiety | 1 (16.67) | 0 (0) | 6 (28.57) | 1 (20) | 0 (0) | 0.543 |
| Numbness | 0 (0) | 0 (0) | 2 (9.52) | 0 (0) | 1 (14.29) | 0.928 |
| Sleep problems | 1 (16.67) | 2 (50) | 7 (33.33) | 2 (40) | 2 (28.57) | 0.919 |
| Palpitation | 1 (16.67) | 0 (0) | 8 (38.10) | 2 (40) | 3 (42.86) | 0.613 |
| Dysosmia or anosmia | 2 (33.33) | 1 (25) | 4 (19.05) | 1 (20) | 1 (14.29) | 0.279 |
| More than 2 organs involved | 2 (33.33) | 1 (25) | 12 (57.14) | 3 (60) | 4 (57.14) | 0.712 |

&Disease activity was evaluated according to Kerr criteria within three months before COVID-19 infection. #The patients also suffered systemic symptoms, such as fever, fatigue, and/or myalgia. GCs, Glucocorticoids; bDMARDs, biologic disease-modifying anti-rheumatic drugs; csDMARDs, conventional synthetic disease-modifying anti-rheumatic drugs.

**Supplemental Table S3**. Comparison of clinical features between TAK patients with persistent active disease and patients who entered remission prior to COVID-19 infection

| **Characteristics** | **Activity& after COVID-19**  **(n=7)** | **Inactivity after COVID-19**  **(n=11)** | ***p*** |
| --- | --- | --- | --- |
| Age (mean±SD, years) | 40.43±15.69 | 38±10.33 | 1 |
| Female, n (%) | 7 (100) | 8 (72.73) | 0.245 |
| BMI (mean±SD) | 22.02±2.82 | 25.32±6.29 | 0.258 |
| Smokers, n (%) | 0 | 0 | / |
| Disease duration (median, IQR, months) | 32.5 (2.5, 214.75) | 12 (4, 60) | 0.580 |
| COVID-19 vaccinated patients, n (%) | 5 (71.43) | 7 (63.64) | 1 |
| Duration of COVID-19 infection (median, IQR, days) | 9 (8, 10) | 7 (6, 10) | 0.142 |
| Interval from COVID-19 infection to the first follow-up, (median, IQR, days) | 59 (54, 74.5) | 53 (30.5, 80) | 0.548 |
| Imaging type, n (%) |  |  |  |
| I | 0 (0) | 1 (9.09) | 1 |
| IIA or IIB | 2 (28.57) | 1 (9.09) | 0.528 |
| III | 0 (0) | 0 (0) |  |
| IV | 0 (0) | 1 (9.09) | 1 |
| V | 5 (71.43) | 8 (72.73) | 1 |
| TAK-associated ischemic event, n (%) | 3 (42.86) | 7 (63.64) | 0.630 |
| Renal hypertension or renal atrophy | 1 (14.29) | 5 (45.45) |  |
| Cerebral infarction | 1 (14.29) | 2 (18.18) |  |
| Pulmonary hypertension | 2 (28.57) | 0 (0) |  |
| Heart failure or cardiac infarction | 1 (14.29) | 0 (0) |  |
| Major treatment after COVID-19 infection, n (%) |  |  |  |
| GCs and bDMARDs | 2 (28.57) | 5 (45.45) | **0.04** |
| GCs and JAK inhibitors | 1 (14.29) | 3 (27.27) |  |
| GCs and csDMARDs | 2 (28.57) | 2 (18.18) |  |
| Single GCs | 0 (0) | 1 (9.09) |  |
| No treatment | 2 (28.57) | 0 (0) |  |
| COVID-19 infection, n (%) | 7 (100) | 11 (100) |  |
| Fever | 5 (71.43) | 8 (72.73) | 1 |
| Highest body temperature（mean±SD, ℃） | 38.82±0.66 | 38.76±0.93 | 0.826 |
| Fatigue | 5 (71.43) | 7 (63.64) | 1 |
| Myalgia | 6 (85.71) | 5 (45.45) | 0.151 |
| Cough | 5 (71.43) | 7 (63.64) | 1 |
| Chest discomfort | 2 (28.57) | 2 (18.18) | 1 |
| Pharyngitis | 4 (57.14) | 2 (18.18) | 0.141 |
| Gastrointestinal symptoms | 0 (0) | 1 (9.09) | 1 |
| Dysosmia or anosmia | 4 (57.14) | 5 (45.45) | 1 |
| Joint pain | 3 (42.86) | 4 (36.36) | 1 |
| Headache | 5 (71.43) | 4 (36.36) | 0.335 |
| Sleep problems | 0 (0) | 5 (45.45) | 0.101 |
| More than 2 organs involved | 5 (71.43) | 6 (54.55) | 0.637 |
| Remaining symptoms after 30 days of COVID-19 infection, n (%) | 2 (28.57) | 1 (9.09) | 0.528 |

&Disease activity was evaluated according to Kerr criteria three months after COVID-19 infection. #Patients also suffered systemic symptoms, such as fever, fatigue, and/or myalgia. GCs, glucocorticoids; bDMARDs, biologic disease-modifying anti-rheumatic drugs; csDMARDs, conventional synthetic disease-modifying anti-rheumatic drugs; JAK, Janus kinase.

**Supplemental Table S4.** Clinical differences in new-onset flare TAK patients with and without COVID-19 infection

|  | **Infected group**  **(n=14)** | **Uninfected group**  **(n=11)** | p |
| --- | --- | --- | --- |
| Age (mean±SD, years) | 37.86±11.79 | 33.73±12.21 | 0.351 |
| Female, n (%) | 13 (92.86) | 8 (72.73) | 0.288 |
| BMI (mean±SD) | 24.30±4.45 | 22.52±2.30 | 0.460 |
| Smoker, n (%) | 1 (7.14) | 1 (9.09) | 1 |
| Disease duration (median, IQR, months) | 60 (15, 120) | 36 (12, 60) | 0.350 |
| COVID-19 vaccinated patients, n (%) | 7 (50) | 5 (45.45) | 0.821 |
| Duration of COVID-19 infection (median, IQR, days) | 7 (5, 8.75) | / |  |
| Duration of first visit to hospital after COVID-19 infection, (median, IQR, days) | 71 (19.25, 85.5) | 79 (46, 89) | 0.427 |
| Imaging type, n (%) |  |  |  |
| I | 7 (50) | 5 (45.45) | 0.821 |
| IIA or IIB | 1 (7.14) | 2 (18.18) | 0.565 |
| III | 0 (0) | 0 (0) | / |
| IV | 1 (7.14) | 0 (0) | 1 |
| V | 5 (35.71) | 4 (36.36) | 1 |
| TAK-associated organ involvement, n (%) | 5 (35.71) | 5 (45.45) | 0.697 |
| Renal hypertension or renal atrophy | 2 (14.29) | 1 (9.09) | 1 |
| Cerebral infarction | 1 (7.14) | 2 (18.18) | 0.565 |
| Pulmonary hypertension | 0 (0) | 1 (9.09) | 0.440 |
| Heart failure or cardiac infarction | 2 (14.29) | 1 (9.09) | 1 |
| Major treatment, n (%) |  |  | 0.19 |
| GCs and bDMARDs | 2 (14.29) | 2 (18.18) | 1 |
| GCs and JAK inhibitors | 1 (7.14) | 2 (18.18) | 0.565 |
| GCs and csDMARDs | 10 (71.43) | 3 (27.27) | **0.028** |
| Single GCs | 0 (0) | 2 (18.18) | 0.183 |
| No treatment | 1 (7.14) | 2 (18.18) | 0.565 |
| TAK-associated symptoms, n (%) |  |  |  |
| Inflammatory-associated ache | 3 (57.14) | 2 (18.18) | 1 |
| Ischemic symptoms | 7 (50) | 4 (36.36) | 0.689 |
| Laboratory indexes |  |  |  |
| ESR | 35.90±22.49 | 40.11±14.24 | 0.513 |
| CRP | 15 (2.95, 37.45) | 7.40 (1.20, 18.75) | 0.513 |
| IL-6 | 3.20 (2.10, 15.95) | 3.30 (2.13, 8) | 0.771 |
| TNF-α | 7.94±4.12 | 10.01±4.20 | 0.336 |

GCs, glucocorticoids; bDMARDs, biologic disease-modifying anti-rheumatic drugs; csDMARDs, conventional synthetic disease-modifying anti-rheumatic drugs; JAK, Janus kinase; ESR, erythrocyte sedimentation rate; CRP, C-reactive protein; IL, interleukin; TNF, tumor necrosis factor.

**Supplemental Table S5**. The difference of treatments in TAK patients with and without disease activity after COVID-19 infection

| **Characteristics** | **Activity&**  **(n=21)** | **Inactivity**  **(n=142)** | ***p*** |
| --- | --- | --- | --- |
| Age (mean±SD, years) | 38.71±12.87 | 36.15±10.51 | 0.425 |
| Female, n (%) | 20 (95.24) | 125 (88.03) | 0.473 |
| BMI (mean±SD) | 23.54±4.06 | 22.71±4.04 | 0.348 |
| Disease duration (median, IQR, months) | 50.50 (9, 114) | 52.50 (28.50, 108) | 0.458 |
| COVID-19 vaccinated patients, n (%) | 12 (57.14) | 67 (47.18) | 0.394 |
| Imaging type, n (%) |  |  |  |
| I | 7 (33.33) | 36 (25.35) | 0.439 |
| IIA or IIB | 3 (14.29) | 23 (16.20) | 1 |
| III | 0 (0) | 6 (4.23) | 1 |
| IV | 1 (4.76) | 11 (7.75) | 1 |
| V | 10 (47.62) | 64 (45.07) | 1 |
| TAK-associated organ involvement, n (%) | 8 (38.10) | 57 (40.14) | 1 |
| Renal hypertension or renal atrophy | 3 (14.29) | 26 (18.31) | 1 |
| Cerebral infarction | 2 (9.52) | 13 (9.15) | 1 |
| Pulmonary hypertension | 2 (9.52) | 9 (6.34) | 0.587 |
| Heart failure or cardiac infarction | 3 (14.29) | 15 (10.56) | 0.611 |
| Major treatment, n (%) |  |  |  |
| GCs and bDMARDs | 2 (9.52) | 22 (15.49) | 0.11 |
| GCs and JAK inhibitors | 2 (9.52) | 29 (20.42) |  |
| GCs and csDMARDs | 13 (61.90) | 62 (43.66) |  |
| Single GCs | 0 (0) | 17 (11.97) |  |
| No treatment | 4 (61.90) | 12 (8.45) |  |
| Medication changes during COVID-19 infection, n (%) |  |  |  |
| Stopping/reducing duration, (median, IQR, days) | 14.5 (8.75, 15) | 10 (7, 20) | 0.703 |
| Increased GCs | 4 (19.05) | 7 (4.93) | **0.037** |
| Decreased GCs@ | 0 (0) | 8 (5.63) | 0.598 |
| bDMARDs | 1 (4.76) | 10 (7.04) | 1 |
| JAK inhibitors | 1 (4.76) | 14 (9.86) | 0.695 |
| csDMARDs | 2 (9.52) | 18 (12.68) | 1 |

&Disease activity was evaluated according to Kerr criteria three months after COVID-19 infection; @including 1 case who stopped GCs; GCs, glucocorticoids; bDMARDs, biologic disease-modifying anti-rheumatic drugs; csDMARDs, conventional synthetic disease-modifying anti-rheumatic drugs.


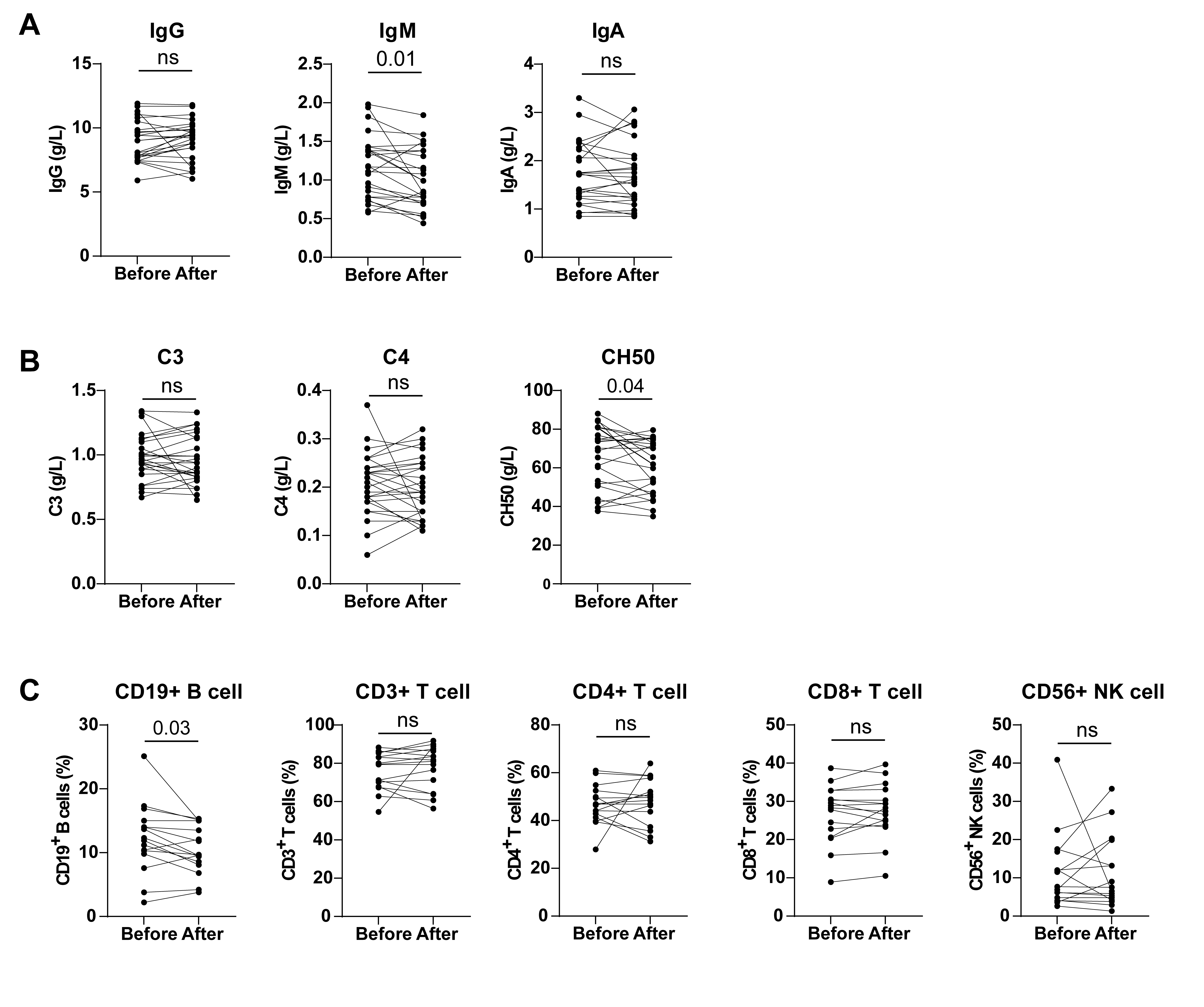


**Supplemental Figure S2**. Changes of immune-related parameters in TAK patients before and after COVID-19 infection. A, changes in immunoglobulins (25 cases); B, changes in complement (25 cases); C, changes in peripheral immune cell percentages (16 cases).
